# Lesion-specific EEG signatures in stroke: a multi-scale framework integrating oscillations, connectivity, and asymmetry for machine learning decoding

**DOI:** 10.1186/s12984-026-01947-2

**Published:** 2026-03-31

**Authors:** Wanting Liu, Conghui Wei, Yajing Lan, Xumiao Peng, Chenyuan Zhai, Zhen Liang, Li Zhang, Yan Gong, Gan Huang

**Affiliations:** 1 School of Biomedical Engineering, Medical School, Shenzhen University, Shenzhen, 518060 Guangdong China; 2 Guangdong Provincial Key Laboratory of Biomedical Measurements and Ultrasound Imaging, Shenzhen, 518060 Guangdong China; 3 Department of Rehabilitation medicine, The Affiliated Suzhou Hospital of Nanjing Medical University, Suzhou, 215000 Jiangsu China; 4 College of Health-Preservation and Wellness, Dalian Medical University, Dalian, 116044 Liaoning China

**Keywords:** Lesion-specific, Stroke, Electroencephalography, Multi-scale analysis, Machine learning

## Abstract

**Background:**

Lesion location is a major source of post-stroke neurophysiological heterogeneity, yet most electroencephalography (EEG) studies analyze patients as a single group, limiting lesion-specific biomarkers and translation. We proposed a lesion-centric, multi-scale EEG framework integrating local oscillations, inter-regional connectivity, and hemispheric asymmetry with machine learning to characterize and decode basal ganglia (P1), fronto-temporal/centrum semiovale (P2), and brainstem (P3) lesions.

**Methods:**

Five-minute eyes-open, 128-channel resting EEG ($1\,\text {kHz}$) was recorded in 57 subacute stroke patients (P1 = 22, P2 = 18, P3 = 17) and 22 matched controls. From artifact-minimized $90\,\text {s}$ segments, ROI-averaged power spectral density (PSD) ($\theta$: 4–$7\,\text {Hz}$; $\alpha$: 7–$12\,\text {Hz}$; $\beta _{1}$: 12–$16\,\text {Hz}$; peak $\alpha$ frequency), current source density (CSD)-based magnitude-squared coherence, and directional BSI (dirBSI) were computed. Between-group and subgroup differences were assessed using *t*-tests/Wilcoxon and ANOVA/Kruskal–Wallis with Benjamini–Hochberg FDR ($q=0.05$). EEG–behavior associations were examined with Spearman correlations. For machine learning, common spatial patterns (CSP) features were classified using linear discriminant analysis (LDA) with leave-one-subject-out cross-validation. To align with clinical workflow, we report HC vs P as “stroke detection/screening” and patient-only P1/P2/P3 classification as “lesion subtype decoding for stratification” (along with pairwise P1 vs P2, P1 vs P3, and P2 vs P3 models). An EEGNet baseline was evaluated for comparison.

**Results:**

Increased $\alpha$ power and a leftward peak $\alpha$ shift were observed in patients (HC: $9.93 \pm 1.09\,\text {Hz}$; P: $8.75 \pm 1.02\,\text {Hz}$; $p = 6.82 \times 10^{-5}$). Pre-FDR, $\theta$-band frontal–motor connectivity was strengthened, while posterior P–O connectivity in $\alpha$/$\beta _{1}$ was weakened. Ipsilesional dominance in $\theta$ was indicated by dirBSI (HC: $-0.026 \pm 0.101$; P: $0.061 \pm 0.122$; $q=0.012$). Across lesions, $\beta _{1}$ power differences in central/parietal/occipital ROIs were detected pre-FDR, with higher parietal $\beta _{1}$ in P3; $\alpha$-band asymmetry was stronger in P1/P2 and more symmetric in P3 ($q=0.028$). EEG–behavior correlations did not survive FDR. Using CSP+LDA, accuracies of 92.41% (HC vs P), 94.87% (P1 vs P3), 85.71% (P2 vs P3), and 82.50% (P1 vs P2) were achieved; all binary AUCs exceeded 0.85; three-class accuracy reached 85.96%.

**Conclusion:**

This multi-scale EEG framework identifies lesion-associated neurophysiological signatures and demonstrates feasible lesion subtype decoding, supporting the potential of EEG biomarkers for objective stratification and precision neurorehabilitation.

**Supplementary Information:**

The online version contains supplementary material available at 10.1186/s12984-026-01947-2.

## Introduction

Stroke remains a leading cause of death and long-term disability worldwide, imposing substantial personal, societal, and economic burdens [1, 2]. Functional impairment after stroke is highly heterogeneous: patients with similar clinical diagnoses can exhibit markedly different motor, cognitive, and daily-life outcomes, as well as distinct recovery trajectories. A major driver of this heterogeneity is lesion location, which shapes the disruption of local circuits and distributed networks and thereby influences post-stroke reorganization and behavioral capacity.

Neuroimaging (computed tomography [CT] / magnetic rsonance imaging [MRI]) provides essential information about lesion anatomy, but it is largely static and does not directly capture time-sensitive changes in neuronal excitability, oscillatory dynamics, and inter-regional communication that emerge during recovery. Electroencephalography (EEG) offers millisecond temporal resolution, bedside feasibility, low cost, and suitability for repeated measurements, making it a practical modality to characterize neurophysiological alterations after stroke and to support monitoring and stratification in neurorehabilitation settings [3]. Quantitative EEG studies have reported clinically relevant spectral and rhythmic changes, including increased low-frequency activity, altered alpha dynamics, and shifts in peak alpha frequency associated with impairment and prognosis [4, 5]. Other work has linked stroke-related disability to disruption of large-scale functional interactions, including resting-state connectivity alterations in motor and associative networks [6, 7]. Collectively, these findings support the utility of EEG for probing post-stroke pathophysiology and recovery.

However, three gaps limit translation toward lesion-informed biomarkers and decision support. First, many EEG studies treat stroke cohorts as homogeneous and focus primarily on the established “patients versus healthy controls” contrast, which is expected given that disrupted cerebral blood flow perturbs neuronal metabolism and excitability. This practice can obscure lesion-dependent mechanisms and reduces interpretability for individualized rehabilitation planning [8, 9]. Second, existing work often emphasizes a single feature family (e.g., band power or connectivity), whereas stroke involves concurrent changes across multiple scales-local oscillations, inter-regional coupling, and hemispheric interactions–that may jointly determine functional status and recovery potential. Third, while machine learning has been increasingly applied in stroke research, EEG-based models are still mostly oriented toward diagnosing stroke or distinguishing patients from controls, with comparatively fewer attempts to decode lesion location in a physiologically interpretable way that could complement imaging and clinical assessment [10, 11]. Methodological challenges further complicate interpretability, including volume conduction effects in scalp-level connectivity and inconsistent treatment of hemispheric asymmetry metrics across studies [12, 13].

In this context, we position the present work as an exploratory lesion-stratified EEG study. While differences in resting-state EEG between stroke patients and healthy individuals are expected, the exploratory contribution of this work is to move beyond the overall contrast and test whether lesion location is associated with separable, multi-scale EEG phenotypes–spanning oscillatory power, inter-regional coherence, and hemispheric asymmetry–that can support lesion-informed characterization and decoding. We adopt a lesion-centric design to reduce mechanistic ambiguity and to encourage hypotheses linked to neuroanatomical systems, while acknowledging the indirect sensitivity of scalp EEG to deep structures and the potential influence of network-level effects [14].

We focused on three lesion subgroups: basal ganglia (P1), fronto-temporal/centrum semiovale (P2), and brainstem (P3). This choice was driven by both clinical relevance and cohort feasibility. Clinically, these locations represent common topographic patterns encountered in routine stroke practice and correspond to partly distinct functional systems [15]. Methodologically, these categories were among the most prevalent in our hospital-based cohort, enabling subgroup sizes that are feasible for exploratory comparisons while maintaining relative anatomical coherence. We therefore restricted lesion-location analyses to these three categories to minimize heterogeneity arising from mixed, multifocal, or rare lesion patterns and to keep subgroup analyses interpretable and tractable at the current sample size.

A key consideration is how lesions in subcortical nuclei or the brainstem may affect scalp-recorded EEG. Although scalp EEG has limited direct sensitivity to deep generators, deep and subcortical lesions can alter cortical dynamics through network-level mechanisms. Basal ganglia lesions can perturb cortico-basal ganglia-thalamo-cortical loops and influence sensorimotor rhythm modulation and interhemispheric balance, potentially manifesting as changes in alpha/beta power and hemispheric asymmetry [8, 16]. Fronto-temporal and centrum semiovale lesions are likely to affect long-range cortico–cortical and cortico–subcortical pathways and may therefore more prominently impact inter-regional coupling patterns, including fronto-motor and fronto-parietal interactions [7]. Brainstem lesions may influence global cortical activation and state regulation via disruption of ascending arousal and neuromodulatory systems, potentially producing more diffuse alterations and distinct hemispheric balance profiles [17]. Based on this lesion-informed rationale, we formulated directional hypotheses that: (H1) basal ganglia lesions (P1) would show more pronounced alterations in sensorimotor-related rhythms and hemispheric imbalance; (H2) fronto-temporal/centrum semiovale lesions (P2) would show relatively stronger disruption of inter-regional coupling; and (H3) brainstem lesions (P3) would show comparatively diffuse changes related to arousal/neuromodulatory pathways and potentially different symmetry profiles. We emphasize that these hypotheses concern the relative prominence of feature families rather than a one-to-one mapping between a lesion site and a single EEG marker, given inter-individual variability in lesion extent and secondary network effects.

To test these hypotheses within an integrated framework, we performed resting-state high-density EEG analyses at three complementary scales: (i) local oscillations quantified by power spectral density (PSD) features, including band-limited power and peak alpha frequency; (ii) inter-regional functional connectivity quantified by magnitude-squared coherence, with current source density (CSD) preprocessing to mitigate volume conduction; and (iii) hemispheric asymmetry quantified by the directional brain symmetry index (dirBSI) [13, 18–20]. We then assessed whether these multi-scale features support lesion decoding using an interpretable machine learning approach based on common spatial patterns (CSP) and linear discriminant analysis (LDA), evaluated with leave-one-subject-out cross-validation (LOSOCV), and compared this feature-engineered strategy with an end-to-end deep learning baseline (EEGNet) [21–23]. Importantly, to better match real-world clinical workflow, we separated two related tasks: HC vs. stroke patients (as a screening/detection benchmark) and patient-only lesion subtype decoding (P1/P2/P3) for stratification after stroke is established; therefore, we primarily report the three-class patient model and pairwise patient comparisons, while using HC vs. P as a complementary validation of stroke–control separability. In addition, we explored associations between EEG features and standardized behavioral measures to examine potential translational relevance.

In summary, this study aims to: (1) characterize multi-scale resting-state EEG differences between subacute stroke patients and healthy controls; (2) investigate whether three clinically prevalent lesion locations (P1/P2/P3) exhibit separable EEG phenotypes in oscillations, connectivity, and hemispheric asymmetry; (3) explore EEG-behavior relationships as an initial step toward translational biomarkers; and (4) evaluate the feasibility of lesion decoding from EEG using interpretable machine learning. By systematically integrating oscillatory, connectivity, and asymmetry features within a lesion-stratified design, our work seeks to bridge mechanistic understanding and clinically actionable stratification, while explicitly recognizing the exploratory nature of lesion-specific EEG profiling and motivating future validation in larger, multi-center cohorts with more fine-grained lesion mapping.

## Materials and methods

### Study design and analysis overview

High-density EEG was recorded for 5 min during rest with eyes open, and subjects were grouped into three subtypes of lesion (P1 / P2 / P3) and healthy controls (HC) based on imaging. Feature extraction targeted three levels: (1) local oscillations using PSD ($\theta$, $\alpha$, $\beta _{1}$, peak $\alpha$ frequency); (2) inter-regional connectivity using coherence with CSD-preprocessed signals; (3) hemispheric asymmetry using the dirBSI. Group comparisons employed parametric and non-parametric tests with false discovery rate (FDR) correction, and correlations were assessed by Spearman rank [24]. For machine learning, spatial features were derived with CSP and classified using LDA with LOSOCV. Binary (HC vs P; P1 vs P2; P1 vs P3; P2 vs P3) and multi-class (P1 vs P2 vs P3) evaluations were performed, reporting accuracy, area under the receiver operating characteristic curve (AUC), and confusion matrices [21, 22, 25, 26]. This workflow links physiological interpretability with discrimination performance for lesion-specific markers.

### Participants

**Fig. 1:**
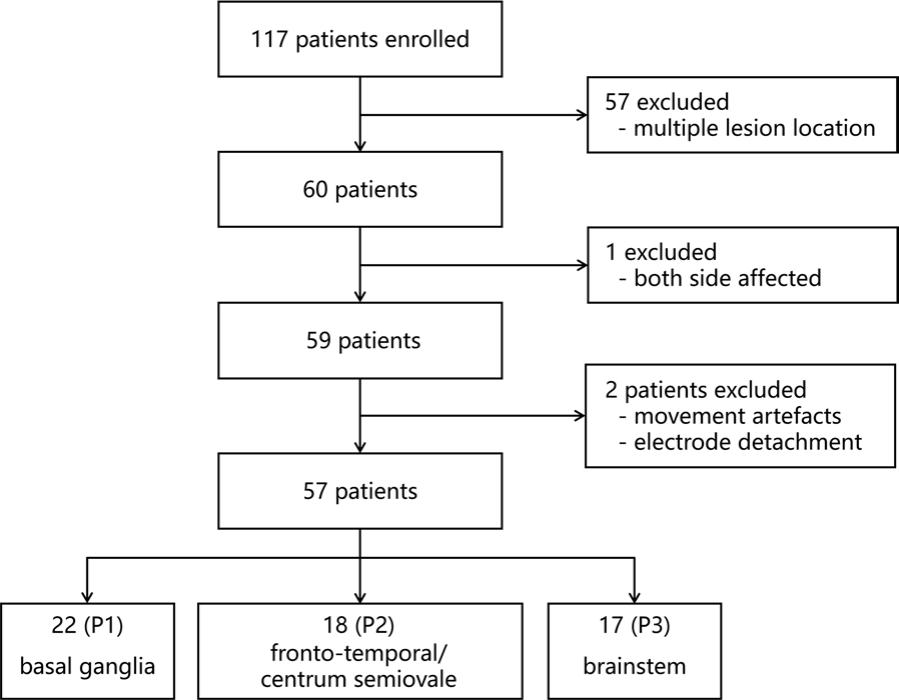
Flowchart of patient enrollment. P1: basal ganglia group; P2: frontal-temporal/centrum semiovale group; P3: brainstem group

A total of 154 participants were initially recruited, including 117 individuals with stroke and 37 healthy controls. Following the screening procedure (see Fig. 1), fifty-seven patients with unilateral stroke accompanied by hemiparesis (41 males; mean age = 60.5 years, SD = 12.0) were included in the final analysis. 22 healthy controls (11 males; mean age = 55.1 years, SD = 9.2) were enrolled using a demographic matching strategy to the patient group. Healthy participants were matched to patients in terms of age distribution and sex, resulting in no statistically significant between group differences. The study protocol was approved by the Institutional Review Board of Suzhou Municipal Hospital (Ethics approval number: K-2024-014-K01). Written informed consent was obtained from participants or their legally authorized representatives when necessary.

Inclusion criteria for stroke patients were as follows: (1) stroke confirmed by CT or MRI, with diagnosis meeting the consensus of the *Diagnostic criteria of cerebrovascular diseases in China (version 2019)*; (2) subacute phase, defined as 2 weeks to 6 months post-onset; (3) upper limb and hand motor impairment, with a Fugl-Meyer Assessment (FMA) wrist-hand subscore $< 24$; (4) age $18 \le \text {years} \le 80$; (5) absence of significant cognitive impairment, with a Mini-Mental State Examination (MMSE) score $\ge 9$.

Exclusion criteria for stroke patients were as follows: (1) history of severe cardiac, pulmonary, hepatic, or renal dysfunction; uncontrolled hypertension, arrhythmia, severe coronary heart disease, or poorly controlled diabetic complications; (2) history or family history of epilepsy; (3) presence of intracranial or extracranial metallic implants; (4) implanted medical devices such as pacemakers or infusion pumps; (5) inability to complete the treatment protocol or poor compliance; (6) pregnancy; (7) participation in other clinical trials in the recent past.

Inclusion criteria for healthy controls were as follows: (1) no history of psychiatric or neurological disorders; (2) no long-term medication with psychotropic drugs; (3) normal or corrected-to-normal vision to ensure task completion. Exclusion criteria for healthy controls were identical to those for the patient group.

Stroke patients were further stratified into three subgroups according to lesion location: basal ganglia group ($n=22$), fronto-temporal/centrum semiovale group ($n=18$), and brainstem group ($n=17$), hereinafter referred to as group P1, P2, and P3, respectively. This location-based stratification was defined a priori to enable an exploratory test of lesion-dependent EEG phenotypes while maintaining cohort feasibility. We restricted lesion-location comparisons to sites with adequate sample size and relatively coherent neuroanatomical substrates; other less frequent or highly heterogeneous lesion locations were not considered in the present lesion-stratified analysis.

### Experimental protocol

Stroke patients underwent two experimental sessions. At hospital admission, MRI or CT was performed to localize the lesion. Transcranial Magnetic Stimulation (TMS) was used to determine motor-evoked potential (MEP) status. During the same session, 5 min of eyes-open resting-state EEG and behavioral assessments were acquired. 16 patients discontinued EEG recording prematurely due to physical discomfort. The second session was conducted at hospital discharge and comprised behavioral assessments only. Healthy controls completed a single session, consisting of 5 min of eyes-open resting-state EEG.

For subsequent analyses, 90 s of minimally contaminated EEG segments were extracted from each patient and control. The inspection focused on identifying and excluding segments contaminated by ocular movements, muscle activity, motion artifacts, amplifier saturation, or non-physiological patterns. Segments exceeding an amplitude threshold of $\pm 100\,\mu \text {V}$ or displaying abnormal spectral characteristics were removed.

### Behavioral measures

A series of standardized behavioral assessment instruments were employed to systematically quantify motor function, cognitive performance, and activities of daily living. All procedures were conducted in accordance with internationally recognized protocols and administered by a rehabilitation therapist with more than five years of clinical experience. Each assessment was performed three times, and the mean score was used for analysis to reduce subjective bias. Prior to testing, participants received detailed instructions to ensure clear understanding of the tasks and optimal compliance during assessment.

- Manual Muscle Testing (MMT). MMT is used as the clinical gold standard for muscle strength evaluation due to its ease of administration, independence from specialized equipment, and strong ecological validity [27]. The scale categorizes muscle strength into six grades (0–5) based on the force generated during muscle contraction and the corresponding joint range of motion. In this study, assessments were focused on the wrist-hand of affected upper limbs.
- FMA. FMA is one of the most commonly employed scales for quantifying post-stroke motor impairment [28]. In this study, we used the Upper Extremity Fugl-Meyer Assessment (UE-FMA), which focuses on the affected upper limb and consists of 33 items across four subdomains: shoulder/elbow/forearm (0–36 points), wrist (0–10 points), hand (0–14 points), and coordination/speed (0–6 points). The total UE-FMA score ranges from 0 to 66, with higher scores reflecting better motor recovery and functional performance. Additionally, the wrist-hand subscore (combining wrist and hand subdomains, 0–24 points) was specifically used for inclusion criteria and further analysis to assess fine motor impairment in the distal upper limb.
- MMSE. MMSE originally developed by Folstein and colleagues in 1975 [29], is a widely applied cognitive screening tool characterized by high sensitivity and brief administration time. It consists of 30 items covering five cognitive domains: orientation, memory, attention and calculation, language, and visuospatial ability. The total score ranges from 0 to 30, with higher scores indicating better cognitive function.
- Activities of Daily Living (ADL). Functional independence was assessed using the modified Barthel Index [30], a standardized instrument widely recognized for its utility in predicting rehabilitation outcomes and informing care planning [31]. The index comprises 10 items encompassing basic activities of daily living and instrumental activities of daily living. The total score ranges from 0 to 100, with five graded levels of independence.

### EEG acquisition

EEG data were collected in a dedicated laboratory from both stroke patients and healthy controls, following identical acquisition and analysis procedures across groups. For all participants, 5 min of resting-state EEG were recorded using a 128-channel EEG system with a Geodesic Sensor Net (EGI Inc., Eugene, OR, USA) at a sampling rate of $1\,\text {kHz}$, and the reference channel was Cz. During recording, electrode impedance was maintained below $20\,\textrm{k}\Omega$, and participants were seated comfortably in a quiet room. Prior to data collection, participants were instructed to remain relaxed, keep their eyes open, fixate on a centrally presented point at eye level, refrain from speaking or moving, and not engage in any intentional cognitive or mental tasks.

### EEG preprocessing and analysis

#### EEG preprocessing

All EEG data were preprocessed using a standardized pipeline. First, a $50\,\text {Hz}$ notch filter was applied to suppress power-line interference. The data were then band-pass filtered between 0.5 and $30\,\text {Hz}$ using a fourth-order Butterworth filter. Ocular artifacts were identified and removed using independent component analysis (ICA). Subsequently, the EEG signals were re-referenced to the common average of all channels. To address the heterogeneity in lesion laterality, a channel-flipping procedure was implemented. Data from patients with left-hemisphere lesions were mirrored to the right hemisphere, ensuring that all patient data were aligned to a right-hemisphere lesion model for the final analysis.

ROIs were defined as follows: frontal region (F: E4, E5, E10, E11, E12, E16, E18, E19), affected motor region (AM: E34, E35, E36, E39, E40, E41, E45, E46), central region (C: E7, E31, E55, E80, E106), unaffected motor region (UM: E102, E103, E104, E108, E109, E110, E115, E116), parietal region (P: E61, E62, E67, E72, E77, E78), and occipital region (O: E70, E71, E74, E75, E76, E81, E82, E83). After hemispheric flipping (patients with left-hemisphere lesions only), AM and UM correspond to the ipsilesional and contralesional motor ROIs, respectively. For each ROI, local PSD, inter-regional functional connectivity, and hemisphere BSI were computed within the $\theta$ (4–$7\,\text {Hz}$), $\alpha$ (7–$12\,\text {Hz}$), and $\beta _{1}$ (12–$16\,\text {Hz}$) bands. The signals within each ROI were averaged and used for subsequent PSD and functional connectivity analyses.

**Fig. 2:**
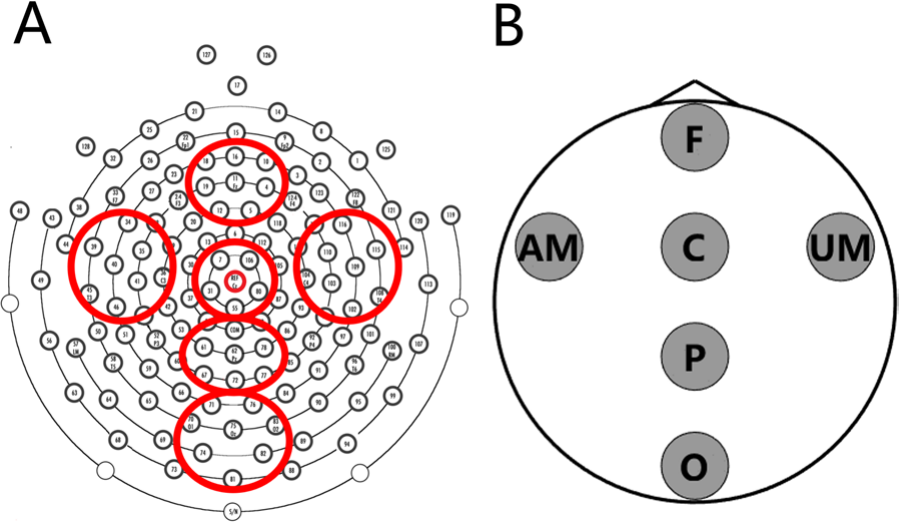
Electrode Layout of the EEG Cap and Definition of ROIs, panel A illustrates the planar layout of the 128-channel EEG cap electrodes. The electrodes circled in red correspond to the regions of interest shown in panel B. F denotes the frontal region; AM, the affected motor region; C, the central region; UM, the unaffected motor region; P, the parietal region; O, the occipital region

#### Power spectral density (PSD)

For all PSD analyses, absolute band power was computed by integrating the PSD within each frequency band over the analyzed frequency range (i.e., without normalization to total power). The local regional PSD was estimated using the Welch method. The continuous signal was divided into overlapping segments, a Hamming window was applied to each segment, and segment-wise periodograms were computed and averaged to reduce variance. A window length of $2\,\text {s}$ with a step size of $1\,\text {s}$ was used for the time-to-frequency transformation. PSD values represent absolute power and were log-transformed into decibel ($\text {dB}$) units using Eq. 1 to improve normality and facilitate statistical analysis. Although CSD transformation can improve spatial specificity, it may attenuate broadly distributed oscillatory power and reduce signal-to-noise ratio in resting-state PSD estimation. Therefore, PSD was computed on average-referenced signals, whereas CSD was applied only in the connectivity analyses, where mitigating volume conduction is critical.

1 $$\begin{aligned} Y = 10 \cdot \log _{10}X \end{aligned}$$

in which *X* denotes the linear-scale PSD in units of ($\mu \text {V}^2/\text {Hz}$), and *Y* denotes the PSD in units of dB.

#### Functional connectivity

To reduce the influence of volume conduction and reference effects on connectivity estimates, we applied a CSD (surface Laplacian) transform using the CSD toolbox [18, 19]. Specifically, the CSD transformation matrices (G and H) were computed for the resulting montage using spherical splines with interpolation constant $m=4$, and the transform was applied with smoothing parameter $\lambda =1\times 10^{-5}$. The head radius was left at the toolbox default (unit sphere), as magnitude-squared coherence is scale-invariant with respect to global amplitude scaling. Functional connectivity was then quantified as magnitude-squared coherence within the frequency bands of interest, using Eq. 2.

2 $$\begin{aligned} C_{XY}(f) = \frac{|P_{XY}(f)|^2}{P_{XX}(f) \cdot P_{YY}(f)} \end{aligned}$$

in which *X* and *Y* denote distinct brain regions, $|P_{XX}(f)|$ and $|P_{YY}(f)|$ are the auto-power spectral densities of the respective regions, and $|P_{XY}(f)|$ is the cross-PSD between them. The coherence coefficient ranges from 0 to 1, with larger values indicating stronger inter-regional coupling.

#### Directional brain symmetry index (dirBSI)

The dirBSI was computed as defined in Eq. 3, by pairing electrodes from the left and right hemispheres in the frequency domain. The BSI was originally defined and subsequently revised by van Putten and Colleagues [20]. Building upon this framework, Saes et al. [13] introduced the dirBSI, enabling the quantification of hemispheric lateralization.

3 $$\begin{aligned} \text {dirBSI}=\frac{\sum _{i = 1}^{n}\sum _{j = 1}^{m}(R_{ij}-L_{ij})}{\sum _{i = 1}^{n}\sum _{j = 1}^{m}(R_{ij}+L_{ij})} \end{aligned}$$

in which $$m = 1, 2, \dots , 59$$ and $$n = 4, 5, \dots , 16$$ denote the channel pairs and frequency bins, respectively; *R* refers to electrodes in the right hemisphere and *L* to electrodes in the left hemisphere. After hemispheric flipping (patients with left-hemisphere lesions only), the lesioned hemisphere is aligned to the right; thus, in patient data *R* and *L* represent the ipsilesional and contralesional hemispheres, respectively. The dirBSI takes values in the range $$[-1, 1]$$, where (0, 1] indicates a predominance of right-hemispheric activity, $$[-1, 0)$$ indicates a predominance of left-hemispheric activity, and 0 indicates symmetric activity between the two hemispheres.

### Statistical analysis

Statistical analyses were performed in MATLAB using two-sided tests with a significance level of $\alpha = 0.05$. To improve clarity and consistency, statistical testing was conducted on ROI- and frequency-band–aggregated features for both PSD and functional connectivity, and on band-level values for hemispheric asymmetry. Specifically, EEG signals were first averaged within each ROI (F, AM, C, UM, P, O; Fig. 2). PSD and coherence were then summarized within three predefined frequency bands ($\theta$, $\alpha$, $\beta _{1}$) prior to statistical testing, whereas dirBSI was computed as a hemispheric-level metric for each band.

Normality of each feature was assessed using the Lilliefors test. Between-group comparisons (HC vs. P) were performed for each feature using an independent-samples *t*-test when normality assumptions were met; otherwise, a Wilcoxon rank-sum test was applied. Differences among the three patient subgroups (P1/P2/P3) were assessed using one-way ANOVA for normally distributed features; otherwise, a Kruskal–Wallis test was used. When the omnibus test was significant, we conducted post hoc pairwise comparisons using parametric or non-parametric tests consistent with the data distribution. When the omnibus test was not significant, any post hoc pairwise analyses were considered exploratory and are reported only as uncorrected trends.

Multiple comparisons were controlled using the Benjamini–Hochberg false discovery rate (FDR) procedure with $q = 0.05$, applied within each analysis family and group contrast. The number of hypothesis tests per contrast was as follows: (1) PSD: band $\times$ ROI = $3 \times 6 = 18$ tests; (2) Functional connectivity: band $\times$ ROI-pairs = $3 \times 15 = 45$ tests (15 unique ROI pairs from 6 ROIs); (3) Hemispheric asymmetry (dirBSI): band = 3 tests.

For transparency, when no effects survived FDR correction, we report uncorrected *p*-values only as exploratory trends and explicitly label them as such in the Results.

In addition, we performed a targeted follow-up analysis of peak $\alpha$ frequency (PAF) as a physiologically interpretable spectral marker. PAF was extracted at the ROI level from each participant’s PSD and compared between groups using the same inferential framework as above, with FDR correction applied to the set of ROI-level PAF tests. Associations between EEG features and behavioral measures were assessed using Spearman’s rank correlation. FDR correction ($q = 0.05$) was applied within each family of correlation tests [24].

### Machine learning

CSP is a well-established spatial filtering technique extensively applied in EEG analysis [21]. CSP algorithms aim to identify a set of spatial filters that maximize variance differences between classes, thereby enhancing discriminative features embedded in EEG signals. Specifically, multichannel EEG signals are projected onto these optimized spatial filters, and the logarithmic variance of the filtered signals is extracted as feature vectors for subsequent classification tasks [22]. In this study, 128 EEG channels were used, and CSP filters were computed within three frequency bands of interest. For each binary classification task, two spatial filters (corresponding to the largest and smallest eigenvalues) were selected. Covariance matrices were computed at the trial level and normalized across channels for each subject to mitigate the influence of inter-channel energy scale differences. To facilitate neurophysiological interpretation, CSP spatial filters were transformed into spatial patterns, from which topographical maps were generated. Pattern amplitudes were normalized within each frequency band for visualization only.

For feature-based classification, LDA was employed as the classifier. A LOSOCV strategy was adopted to ensure robustness while minimizing subject-specific bias due to the limited sample size. CSP was first applied to perform binary classification between different patient groups. For comparisons involving three patient subgroups, a one-vs-one scheme was adopted to implement three-class classification, where CSP features extracted from each pairwise comparison were concatenated prior to classification [25].

In addition to the CSP-based approach, an end-to-end deep learning framework was implemented using EEGNet, a compact convolutional neural network specifically designed for EEG signal decoding [23]. EEGNet directly operates on minimally processed EEG time-series data and automatically learns temporal, spatial, and spectral representations without explicit feature engineering. The network architecture consisted of temporal convolutions (kernel size $1 \times 64$) for frequency-specific feature extraction, depthwise spatial convolutions across channels, and separable convolutions (kernel size $1 \times 16$) for efficient feature integration, followed by average pooling and dropout (rate = 0.5). EEG signals were z-score normalized at the input layer. The network was trained using the Adam optimizer with an initial learning rate of 0.001. Training hyperparameters were adjusted according to task complexity: for binary classification, a mini-batch size of 8 and a maximum of 30 epochs were used, whereas for multiclass classification, a mini-batch size of 32 and a maximum of 100 epochs were adopted. EEGNet was evaluated using the same LOSOCV strategy to ensure fair comparison with the CSP-based classifier.

Model performance was evaluated using multiple metrics across several binary classification tasks, including HC vs P (P1, P2, and P3), P1 vs P2, P1 vs P3, and P2 vs P3. For each task, the first group was designated as the positive class and the second as the negative class, and the area under the receiver operating characteristic curve (AUC) was computed [26, 32]. The ROC curve illustrates the trade-off between true positive rate (TPR) and false positive rate (FPR) across varying decision thresholds, with higher AUC values indicating superior discriminative performance.

For multi-class classification, confusion matrices were constructed to provide a comprehensive visualization of classification performance [33]. These matrices depict the correspondence between predicted and true labels for each class, enabling detailed examination of misclassification patterns and potential class-specific biases. This unified evaluation framework ensured a systematic and fair assessment of both CSP-based and EEGNet-based classification approaches.

## Results

### Demographics and clinical characteristics

Table 1 summarizes the demographic and behavioral characteristics of healthy participants and stroke patients. No significant differences were found in age, gender, months post-onset, or stroke type among groups (all $p > 0.05$). However, behavioral assessments revealed lesion-specific differences. Patients in group P2 showed more frequent low MMSE scores than those in P1 and P3 ($p = 0.0006$; P1 vs. P2, $p = 0.0048$). Group P1 exhibited lower MMT scores than groups P2 and P3 ($p = 0.0021$), but greater improvement from baseline ($p = 0.0016$; P1 vs. P2, $p = 0.0222$). FMA scores were significantly lower in P1 compared with P2 and P3, both at baseline and follow-up (all $p < 0.01$). Significant group differences were also observed in ADL scores at both time points ($p < 0.05$), with P1 showing lower follow-up scores than P2 and P3 ($p = 0.0018$). No significant differences were detected in the remaining behavioral measures.

**Table 1:** Behavioral scale scores of healthy participants and stroke patients; statistically significant differences are indicated in bold

Group	HC(N=22)	P1(N=22)	P2(N=18)	P3(N=17)	F/H	df	p	P1 vs. P2	P1 vs. P3	P2 vs. P3
Age	55.1$\pm$9.2	57.0$\pm$10.8	61.2$\pm$12.3	64.5$\pm$12.3	2.7240	3	0.0502	–	–	–
Gender (female/male)	11/11	6/16	5/13	5/12	3.3710	3	0.3379	–	–	–
Days Post-onset	–	24.4$\pm$13.6	27.6$\pm$24.6	27.5$\pm$20.5	0.5480	2	0.7604	-	-	-
Affected side (left/right)	–	11/11	8/10	7/10	0.3099	2	0.8564	–	–	–
Stroke type (IS/HS)	–	17/5	12/6	15/2	2.2698	2	0.3215	–	–	–
Behavioral scale										
MMSE (H/L)	–	19/3	5/13	12/5	14.9117	2	**0**.**0006**	**0**.**0048**	1.0000	**0**.**0919**
MMT	–	2.1$\pm$1.2	3.3$\pm$0.7	3.2$\pm$0.9	12.3043	2	**0**.**0021**	**0**.**0076**	**0**.**0243**	1.0000
MMT at follow-up	–	2.9$\pm$0.9	3.4$\pm$0.7	3.5$\pm$0.7	6.5754	2	**0**.**0373**	0.1416	0.1243	1.0000
MMT improvement (yes/no)	–	14/8	3/15	3/16	12.9089	2	**0**.**0016**	**0**.**0222**	0.0653	1.0000
MEP (yes/no)	–	5/17	7/11	7/10	1.8020	2	0.4062	–	–	–
FMA	–	16.7$\pm$5.0	22.2$\pm$5.4	25.4$\pm$4.8	14.7955	2	**<0.0001**	**0**.**0035**	**<0.0001**	0.1594
FMA at follow-up	–	20.2$\pm$5.6	28.3$\pm$6.1	31.5$\pm$4.8	21.8169	2	**<0.0001**	**0**.**0001**	**<0.0001**	0.2270
FMA improvement	–	3.5$\pm$2.0	6.1$\pm$2.3	6.1$\pm$2.6	18.9824	2	**0**.**0001**	**0**.**0011**	**0**.**0008**	1.0000
ADL	–	45.7$\pm$14.7	60.0$\pm$25.3	59.7$\pm$22.9	6.2113	2	**0**.**0448**	0.1164	0.0975	1.0000
ADL at follow-up	–	57.3$\pm$14.0	70.3$\pm$17.9	73.6$\pm$10.1	7.1543	2	**0**.**0018**	**0**.**0175**	**0**.**0027**	0.7759
ADL improvement	–	11.6$\pm$4.2	10.3$\pm$10.5	13.9$\pm$18.3	0.2285	2	0.8920	–	–	–

IS denotes ischemic stroke; HS, hemorrhagic stroke; H, MMSE scores $\ge$ 21; and L, MMSE scores < 21

### Difference between healthy participants and stroke patients

**Fig. 3:**
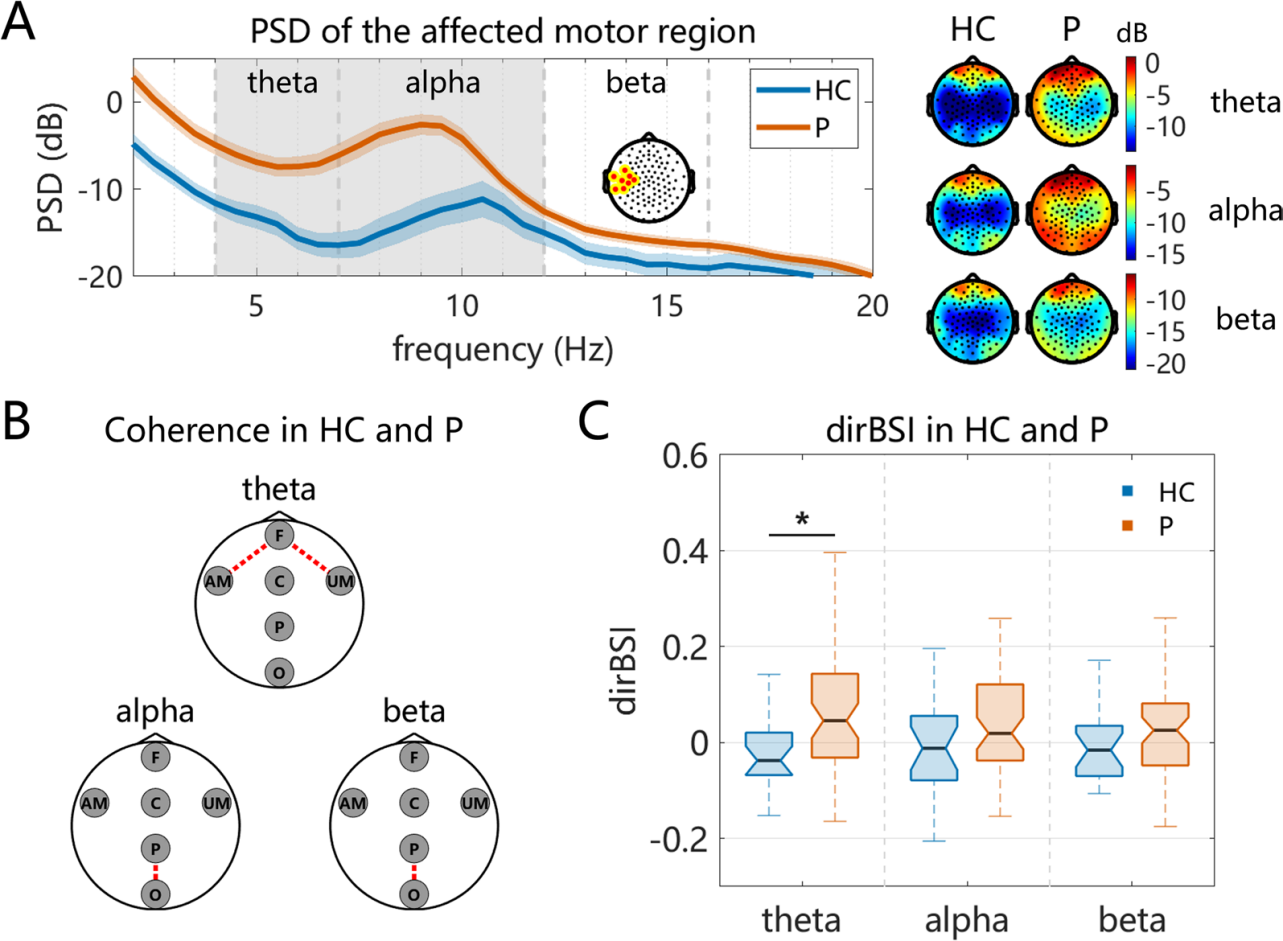
Differences in EEG characteristics between the healthy control (HC) group and the patient (P) group. Panel A shows the PSD of the $\theta$, $\alpha$, and $\beta _{1}$ bands in both groups. Shaded regions around the curves represent the standard error of the mean (SEM). Shaded gray regions indicate frequency ranges showing statistically significant group differences after multiple-comparison correction ($q < 0.05$). The topographic maps on the right display the spatial distribution of these frequency bands for the two groups. Panel B illustrates the significance of region-level connectivity between the HC and P groups, with the dashed line indicating statistical significance ($p < 0.05$, but none survived after FDR correction). Panel C presents box plots of the dirBSI for both groups, where the asterisk (*) denotes statistical significance (FDR-corrected $q < 0.05$)

In the local oscillatory PSD analysis, *t*-tests indicated that, after FDR correction, all regions showed significant differences between patients and healthy controls except F region in the $\alpha$ band and AM, UM, and P region in the $\beta _{1}$ band (all FDR-corrected $q < 0.05$). The largest *t*-value was observed at $\alpha$ band AM region (HC: $-14.89 \pm 7.88\,\text {dB}$; P: $-4.36 \pm 9.10\,\text {dB}$; $t = 4.07$, FDR-corrected $q = 0.001$), as illustrated in Fig. 3A. Further, a significant leftward shift in peak alpha frequency was observed at AM in stroke patients compared with healthy controls (HC: $9.93 \pm 1.09\,\text {Hz}$; P: $8.75 \pm 1.02\,\text {Hz}$; $p = 6.82 \times 10^{-5}$).

In cross-region functional connectivity, no statistically significant results were observed between the two groups after FDR correction. Prior to FDR correction, the patient group exhibited stronger $\theta$ band connectivity between the frontal and motor regions, including F–AM connectivity (HC: $0.100 \pm 0.074$, P: $0.136 \pm 0.102$, $p = 0.032$) and F–UM connectivity (HC: $0.117 \pm 0.071$, P: $0.162 \pm 0.115$, $p = 0.023$). In contrast, the patient group demonstrated weaker P–O connectivity compared with healthy controls in the $\alpha$ band (HC: $0.274 \pm 0.120$, P: $0.222 \pm 0.088$, $p = 0.026$) and $\beta _{1}$ band (HC: $0.225 \pm 0.113$, P: $0.187 \pm 0.088$, $p = 0.044$), as illustrated in Fig. 3B. No significant differences were observed in other ROI-to-ROI coherence values.

For hemisphere BSI analysis, the patient group exhibited an ipsilesional-dominant activity pattern in the $\theta$ band, whereas the healthy control group maintained interhemispheric symmetry (HC: $-0.026 \pm 0.101$, P: $0.061 \pm 0.122$, FDR-corrected $q = 0.012$), as illustrated in Fig. 3C. No significant differences were observed in other frequency domains.

### Differences among patients with different lesion locations

**Fig. 4:**
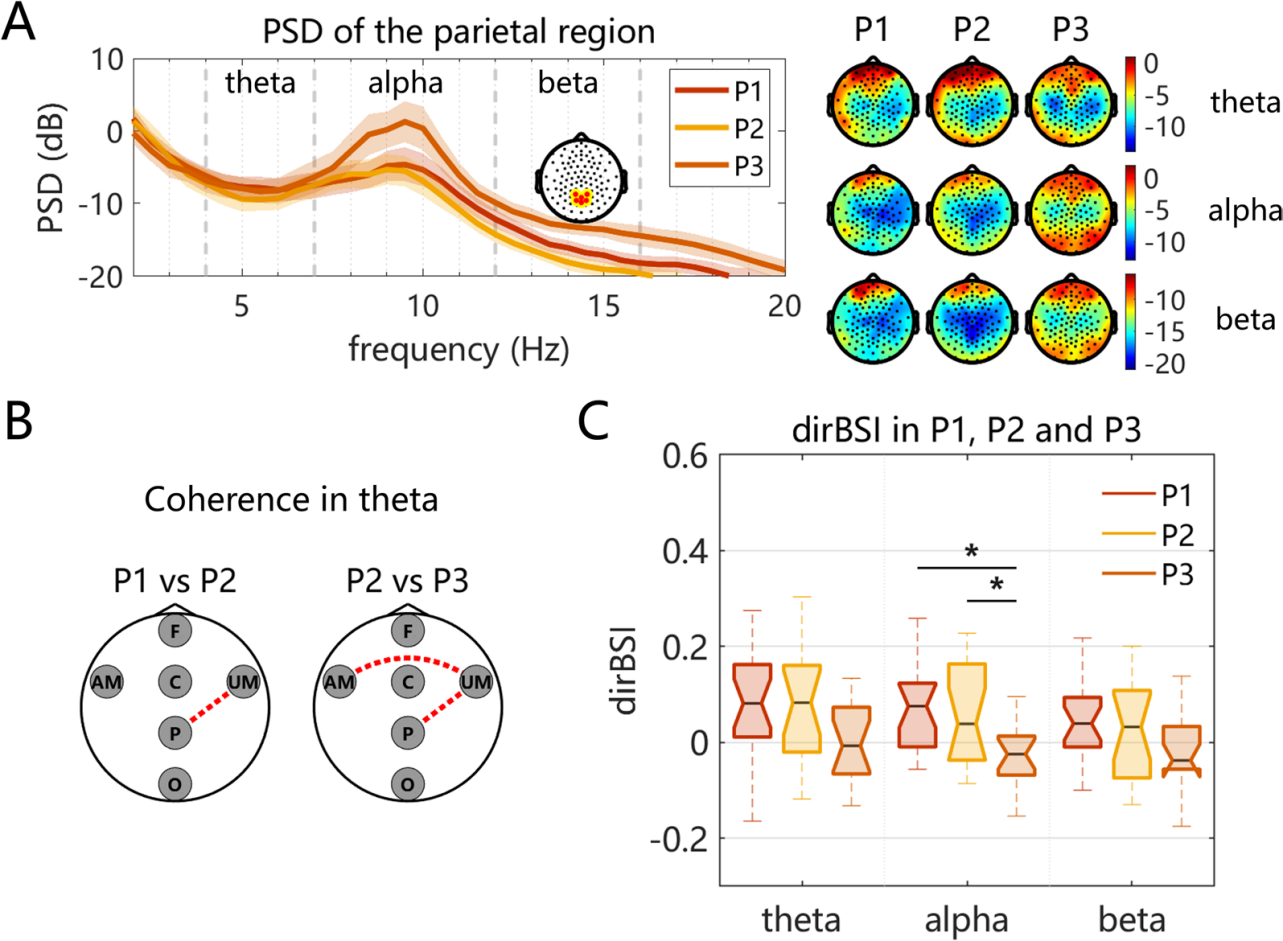
Differences in EEG characteristics among patient subgroups with different lesion locations. Panel A shows the PSD of the $$\theta $$, $$\alpha $$, and $$\beta _{1}$$ bands in each subgroup. Shaded regions around the curves represent the SEM. The topographic maps on the right display the spatial distribution of these frequency bands for the three subgroups. Panel B illustrates the significance of region-level connectivity among group P1, P2 and P3, with the dashed line indicating statistical significance ($$p < 0.05$$, but none survived after FDR correction). Panel C presents box plots of the dirBSI for the three subgroups, where the asterisk (*) denotes statistical significance (FDR-corrected $$ q < 0.05$$)

In the local regional PSD analysis, one-way ANOVA indicated that, after FDR correction, no statistically significant results were observed among the three patient subgroups (all $q > 0.05$). Prior to FDR correction, C, P and O regions showed significant differences in the $\beta _{1}$ band among patient subgroups (all $p < 0.05$). Post-hoc comparisons further showed significant differences between P2 and P3 in C ($p = 0.03$) and P regions ($p = 0.008$) , and between P1 and P3 in the O region ($p = 0.01$). The largest *F*-value was observed at P region among patient subgroups (P1: $-17.27 \pm 4.90\,\text {dB}$, P2: $-19.01 \pm 4.63\,\text {dB}$, P3: $-13.71 \pm 4.75\,\text {dB}$, $F = 4.90$, $p = 0.01$), as illustrated in Fig. 4A. No statistically significant difference was observed in other frequency bands. No significant leftward or rightward shift in peak alpha frequency was observed among patient subgroups.

In cross-region functional connectivity, no statistically significant differences were observed among the three subgroups. Exploratory post hoc pairwise comparisons using the Wilcoxon rank-sum test (uncorrected *p*-values) suggested a $\theta$-band trend in UM–P connectivity, with group P2 showing higher coherence than both P1 and P3 (P1: $0.108 \pm 0.066$, P2: $0.169 \pm 0.109$, P3: $0.114 \pm 0.112$; P1 vs. P2, $p = 0.043$; P2 vs. P3, $p = 0.039$), whereas no difference was observed between P1 and P3. Similarly, AM–UM connectivity showed an exploratory trend for higher coherence in P2 than P3 (P2: $0.150 \pm 0.123$, P3: $0.086 \pm 0.105$, $p = 0.031$), as illustrated in Fig. 4B.

For hemisphere BSI analysis, significant group differences were observed in $\alpha$ band. As illustrated in Fig. 4C, hemispheric activity in groups P1 and P2 was more lateralized toward the contralesional hemisphere, whereas group P3 exhibited a relatively symmetric pattern (P1: $0.068 \pm 0.087$, P2: $0.063 \pm 0.103$, P3: $-0.021 \pm 0.090$, FDR-corrected $q = 0.028$). Post hoc tests further revealed significant differences between group P3 and both groups P1 and P2 ($p < 0.05$). No significant differences were identified in other frequency domains.

### Relationship between EEG and behavioral measures

Associations between EEG features and behavioral outcomes were explored. After FDR correction, no statistically significant correlations remained. Before correction, several associations emerged, for instance, MMT showed positive correlations with $\theta$, $\alpha$, and $\beta _{1}$ band connectivity in certain regions, while cognitive status correlated positively with $\theta$ band connectivity in some networks. However, these effects lacked a consistent or systematic pattern across groups or conditions. Owing to this heterogeneity, full statistical details are provided in the Additional file 1 for clarity and completeness.

### Machine learning

The classification results obtained using CSP-based features and EEGNet are summarized in Table 2. For the CSP-based approach, high classification performance was achieved in distinguishing healthy participants from patients (accuracy = $92.41\%$), as well as in differentiating patient groups P1 and P3 (accuracy = $94.87\%$). In contrast, discrimination between groups P1 and P2 yielded the lowest performance among the binary tasks, although the accuracy remained relatively high (accuracy = $82.50\%$). Furthermore, three-class classification of patients with different lesion locations using a one-vs-one CSP strategy also produced satisfactory results (accuracy = $85.96\%$). To further characterize the discriminative features underlying the CSP-based classification, the spatial patterns of the leading CSP components were examined across different frequency bands and group comparisons (Fig. S1).

**Table 2:** Classification accuracy (%) of CSP-based and EEGNet-based models across binary and multiclass tasks; the better result for each task is highlighted in bold

Class label	HC/P	P1/P2	P1/P3	P2/P3	P1/P2/P3
CSP accuracy	**92**.**41**	**82**.**50**	**94**.**87**	**85**.**71**	**85**.**96**
EEGNet accuracy	75.95	45.00	48.72	37.14	33.33

**Fig. 5:**
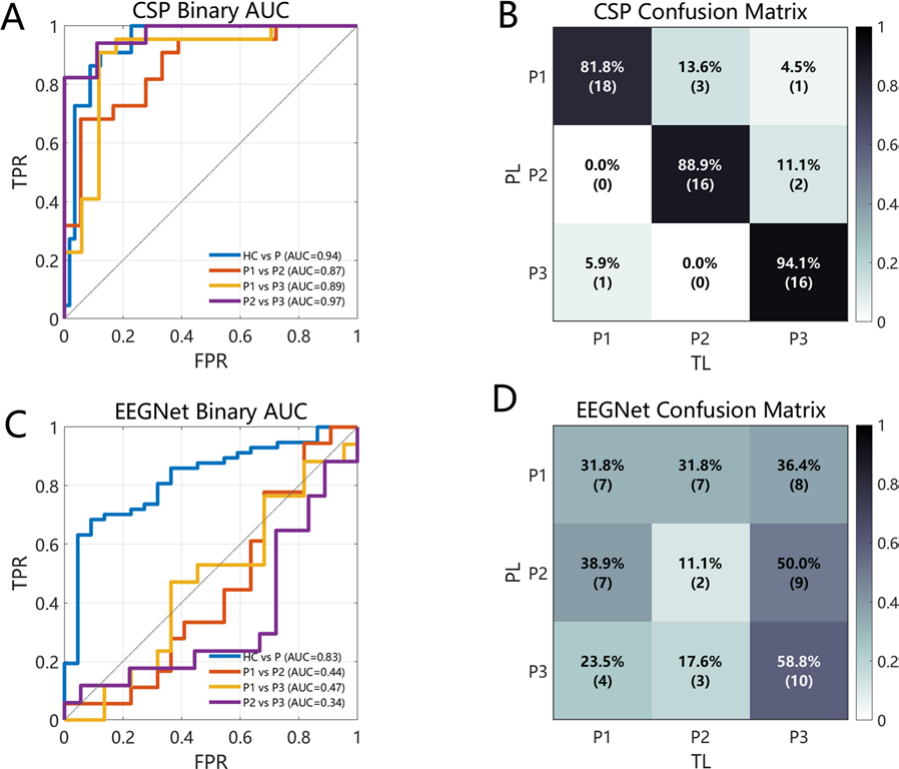
Performance comparison between CSP-based and EEGNet-based classifiers. Panels A and C show the ROC curves for binary classification tasks using CSP and EEGNet, respectively, with the dashed line indicating random guessing (AUC = 0.5). Panels B and D present the normalized confusion matrices for the three-class classification obtained using CSP and EEGNet, respectively, where values indicate the proportion of predictions in each class. TPR denotes true positive rate; FPR, false positive rate. PL represents predicted label; TL, true label

To provide a more comprehensive evaluation, receiver operating characteristic (ROC) curves and corresponding AUC values were computed. As shown in Fig. 5A, all CSP-based binary classification tasks yielded AUC values above 0.85, indicating robust discriminative capability. Notably, the ROC curve for P1 vs P2 was consistently lower than those for other group comparisons, suggesting greater similarity between these two groups. In the three-class setting (Fig. 5B), misclassifications occurred most frequently when the true label was P1, which tended to be confused with both P2 and P3.

For comparison, EEGNet was evaluated under the same LOSOCV framework. As shown in Table 2 and Fig. 5(C,D), EEGNet exhibited substantially lower classification performance across both binary and multiclass tasks. The AUC values for binary classification ranged from 0.34 to 0.83. Notably, EEGNet demonstrated a relatively better ability to discriminate healthy controls from patients, whereas its performance in distinguishing patient groups with different lesion locations was markedly reduced. In the three-class classification task, the overall accuracy was close to chance level (accuracy = $33.33\%$). As illustrated in the confusion matrix (Fig. 5D), EEGNet was able to correctly identify group P3 to some extent (with the TPR = $58.8\%$), while substantial confusion was observed between groups P1 and P2.

## Discussion and conclusion

### Main findings

This study presents a multidimensional resting-state EEG framework that integrates spectral power, inter-regional functional connectivity, and hemispheric symmetry into a unified analytic pipeline for high-density EEG in subacute stroke. Using this framework, we systematically compared patients with three lesion locations–basal ganglia (P1), fronto-temporal/centrum semiovale (P2), and brainstem (P3)–with demographically matched healthy controls (HC). Several findings emerged. First, at the local oscillatory level, patients showed elevated $\alpha$-band power, most prominently in the motor-related region, together with a significant slowing of peak $\alpha$ frequency relative to controls. Second, at the network level, group differences in coherence did not survive FDR correction, but pre-FDR analyses suggested strengthened frontal–motor coupling in $\theta$ and weakened posterior (parietal–occipital) coupling in $\alpha$/$\beta _{1}$ in patients. Third, at the hemispheric level, patients exhibited ipsilesional dominance in $\theta$ as indexed by dirBSI, whereas controls remained largely symmetric. Fourth, lesion-stratified analyses indicated that P3 tended to show higher parietal $\beta _{1}$ power (pre-FDR) and a more symmetric $\alpha$-band dirBSI profile relative to P1/P2, which showed stronger contralesional lateralization. Finally, CSP+LDA achieved high performance in both stroke screening (HC vs P) and lesion subtype decoding (P1/P2/P3), whereas EEGNet performed substantially worse under the same LOSOCV evaluation. Together, these results support the feasibility of lesion-informed EEG profiling and decoding within an interpretable, multi-scale framework.

### Multi-scale EEG alterations after stroke: oscillations, connectivity, and hemispheric balance

The observed EEG alterations are broadly consistent with established neurophysiological responses to cerebral injury, reflecting both disrupted neural dynamics and compensatory reorganization. Locally, the elevated ipsilesional $\alpha$ activity in motor-related regions may indicate a shift toward an inhibitory or idling state, potentially reflecting reduced cortical excitability and altered excitation-inhibition balance during the subacute stage [34]. In parallel, the slowing of peak $\alpha$ frequency is a reproducible marker in neurological conditions and has been linked to changes in cortical rhythm generation and broader alterations in spectral dynamics, which may be relevant to impaired sensorimotor processing and motor execution [5].

At the connectivity scale, although coherence differences did not remain significant after FDR correction, pre-FDR findings suggested a pattern of increased frontal–motor coupling in $\theta$ and reduced posterior coupling in $\alpha$/$\beta _{1}$ in patients. This pattern is compatible with the notion that frontal control systems may be more strongly recruited to support motor function when primary pathways are compromised, while posterior networks may show reduced synchronization reflecting broader network disruption [7]. Importantly, the absence of FDR-surviving connectivity effects also highlights that coherence-based group comparisons can be sensitive to inter-subject variability, multiple-comparison burden, and residual non-neural sources of covariance, even after CSD preprocessing.

At the hemispheric scale, the ipsilesional dominance in $\theta$ captured by dirBSI indicates a frequency-specific hemispheric imbalance after stroke. Given the channel-flipping procedure (lesion aligned to the right hemisphere), positive dirBSI values reflect relatively stronger ipsilesional activity in the analyzed band. Collectively, these oscillatory, network, and symmetry findings reinforce the value of a multi-scale approach: different feature families emphasize complementary aspects of post-stroke physiology and may jointly characterize state changes that are not fully captured by a single metric.

### Lesion-specific signatures and mechanistic considerations

Beyond global patient–control differences, lesion-stratified analyses revealed features suggestive of lesion-location dependence. The brainstem group (P3) showed comparatively higher parietal $\beta _{1}$ power (pre-FDR) and, notably, a more symmetric $\alpha$-band dirBSI profile relative to P1/P2, which exhibited stronger contralesional lateralization. One potential interpretation is that brainstem lesions, by affecting ascending arousal and neuromodulatory pathways, may alter global cortical state regulation in a manner that differs from basal ganglia or fronto-temporal/centrum semiovale lesions, thereby shaping hemispheric balance and the distribution of higher-frequency activity [17]. The increased parietal $\beta _{1}$ activity in P3 may also be compatible with altered thalamocortical drive and frequency-specific network adjustments following sensory or integrative pathway disruption, consistent with frameworks such as thalamocortical dysrhythmia [16].

In contrast, the basal ganglia (P1) and fronto-temporal/centrum semiovale (P2) groups showed more pronounced contralesional $\alpha$-band lateralization, aligning with interhemispheric compensation/imbalance models. This pattern is plausible given that basal ganglia lesions can perturb cortico-basal ganglia-thalamo-cortical loops, and fronto-temporal/centrum semiovale lesions can affect long-range pathways that influence distributed motor and association networks. At the same time, several lesion-subgroup effects did not survive FDR correction, indicating that lesion-specific EEG differences are likely subtle, heterogeneous, and influenced by lesion extent, diaschisis, and individual variability. These results should therefore be interpreted as exploratory and hypothesis-generating rather than definitive lesion biomarkers.

### Translational relevance and interpretation of machine learning results

To evaluate translational potential, we applied machine learning to test whether the extracted EEG information supports clinically relevant classification tasks, including stroke screening (HC vs P) and post-diagnosis lesion subtype decoding (P1/P2/P3). The CSP-based approach achieved high accuracy across tasks, indicating that discriminative multivariate EEG patterns are present and can be captured by an interpretable feature-based model [21, 22]. These decoding results complement the univariate statistical analyses by leveraging multichannel covariance structure, which can contain class-relevant information that may not appear as robust single-feature effects after multiple-comparison correction [35].

In contrast, EEGNet performed substantially worse, especially for lesion subtype decoding. This likely reflects the difficulty of training end-to-end deep learning models in small-sample, subject-level cross-validation settings, where model capacity, inter-subject variability, and limited data per class can hinder generalization [23, 36, 37]. In this context, CSP+LDA may provide a more data-efficient inductive bias by focusing on frequency-specific spatial covariance patterns that are known to be informative in EEG decoding.

To further support physiological interpretability, we visualized the topographical distributions of the leading CSP components (Additional file 1: Fig. S1). For HC versus patients, the CSP patterns were relatively smooth and exhibited a consistent anterior-posterior gradient, suggesting that the classifier primarily exploited large-scale resting-state alterations rather than a single focal contributor. For lesion-subtype comparisons, CSP patterns were less stable and appeared more influenced by individual electrodes. This is not unexpected: CSP is a supervised decomposition optimized for discrimination rather than for isolating a single physiological source, and in heterogeneous clinical cohorts with modest subgroup sizes its spatial patterns can be sensitive to inter-subject variability and residual channel-specific noise [38]. Accordingly, we present the subgroup CSP maps as exploratory visualizations and avoid over-interpreting fine-grained spatial details; larger cohorts and complementary approaches (e.g., source-space modeling or multimodal fusion with imaging) will be important for establishing more robust lesion-specific spatial signatures.

### Relation to prior work and implications for lesion-informed EEG biomarkers

Our findings align with and extend previous EEG studies of stroke by explicitly adopting a lesion-stratified, multi-scale perspective. The observed elevation in $\alpha$-band power and hemispheric asymmetry is consistent with reports linking stroke to altered rhythmic dynamics and interhemispheric imbalance [13, 39]. The pre-FDR pattern of strengthened frontal–motor coupling in $\theta$ is also compatible with prior descriptions of network remodeling and compensatory recruitment after stroke [7]. At the same time, the direction of $\alpha$-power change can vary across studies and stroke stages, and differences between acute and subacute cohorts may reflect time-dependent recovery processes, including changes in inflammation, arousal, and plasticity [40].

Clinically, the present results suggest that integrating oscillatory, connectivity, and hemispheric measures may support lesion-informed electrophysiological profiling. In particular, asymmetry metrics such as dirBSI offer a compact summary of hemispheric balance that may be relevant to neuromodulation planning and longitudinal monitoring. Moreover, the strong performance of CSP-based decoding indicates the feasibility of deriving discriminative signatures from brief resting-state EEG, which could complement imaging by providing dynamic functional information at the bedside. These implications remain preliminary and require validation in larger and more diverse cohorts.

### Limitations and future directions

Several limitations should be considered when interpreting these findings. First, subgroup sample sizes were modest (18–22 participants per lesion category), limiting statistical power and potentially contributing to null findings after multiple-comparison correction, particularly for connectivity analyses. Second, lesion categorization was necessarily coarse; for example, P2 combined fronto-temporal and centrum semiovale lesions, and ischemic and hemorrhagic strokes were analyzed together, which may introduce additional physiological heterogeneity. Third, although groups were demographically matched and no significant between-group differences were observed in age and sex, residual age-related EEG variability and other clinical factors (e.g., lesion volume, medications, sleep quality, fatigue) may still influence EEG patterns. Fourth, while CSD preprocessing was applied to mitigate volume conduction in connectivity analyses, scalp EEG remains an indirect measure of underlying sources, and sensor-level patterns should not be interpreted as precise neuroanatomical localization. Fifth, correlations between EEG features and behavioral scores did not survive FDR correction, suggesting that EEG–behavior relationships may be weaker than expected, non-linear, or confounded by clinical heterogeneity; richer behavioral phenotyping, longitudinal designs, and multivariate modeling may be needed.

Future work should prioritize multi-center studies with larger cohorts and longitudinal measurements across acute, subacute, and chronic stages to characterize temporal trajectories of oscillations, connectivity, and asymmetry. More precise imaging-guided lesion mapping and stratification (including separation of ischemic vs hemorrhagic stroke and quantification of lesion extent) would help reduce heterogeneity and strengthen mechanistic interpretation. Methodologically, incorporating source-space analysis, graph-theoretic network measures, and multimodal integration with structural and functional imaging may improve sensitivity to lesion-specific network disruptions. Finally, although $\delta$ activity has been linked to structural damage after stroke, we excluded $\delta$ in this exploratory study due to its sensitivity to vigilance fluctuations and non-neural artifacts; future studies with stricter control of vigilance and artifacts may help clarify the contribution of $\delta$-band dynamics [41, 42].

### Conclusion

In conclusion, this study provides an integrated, multi-scale framework for lesion-informed characterization of resting-state high-density EEG in subacute stroke. By combining oscillatory power, coherence-based connectivity (with CSD preprocessing), and hemispheric symmetry (dirBSI), we identified global stroke-related alterations and exploratory lesion-dependent signatures, including distinct asymmetry profiles across lesion locations. Moreover, CSP-based machine learning achieved high accuracy for both stroke screening and lesion subtype decoding, supporting the feasibility of interpretable EEG-based stratification. These findings motivate further validation in larger, longitudinal, and multi-center cohorts, with more precise lesion mapping and advanced modeling, to establish robust EEG biomarkers for precision neurorehabilitation.

## Data Availability

The data that support the findings of this study are available from the corresponding author upon reasonable request.

## Supplementary Informtion

Below is the link to the electronic supplementary material. Supplementary file 1 (pdf 1110 KB)

## Abbreviations

CT: Computed tomography
MRI: Magnetic resonance imaging
EEG: Electroencephalography
BCI: Brain-computer interface
PSD: Power spectral density
CSD: Current source density
CSP: Common spatial pattern
LDA: Linear discriminant analysis
FMA: Fugl–Meyer assessment
MMSE: Mini-Mental State Examination
ADL: Activities of daily living
MMT: Manual muscle testing
MEP: Motor-evoked potential
ROI: Region of interest
dirBSI: Directional brain symmetry index
FDR: False discovery rate
HC: Healthy control
P1/P2/P3: Patient subgroups (lesion in Basal Ganglia, Fronto-temporal/Centrum Semiovale, Brainstem)
IS: Ischemic stroke
HS: Hemorrhagic stroke
AUC: Area under the curve
ROC: Receiver operating characteristic
F/AM/UM/C/P/O: Abbreviations for ROIs (Frontal, Affected Motor, Unaffected Motor, Central, Parietal, Occipital)
LOSOCV: Leave-One-Subject-Out Cross-Validation
PAF: Peak alpha frequency

## References

[1] Feigin VL, Brainin M, Norrving B, Martins SO, Pandian J, Lindsay P, et al. World Stroke Organization: global stroke fact sheet. Int J Stroke. 2025;20(2):132–44. 39635884 10.1177/17474930241308142 PMC11786524

[2] Saini V, Guada L, Yavagal DR. Global epidemiology of stroke and access to acute ischemic stroke interventions. Neurology. 2021;97(20 Supplement 2):6–16. 10.1212/WNL.0000000000012781 34785599

[3] Wu J, Srinivasan R, Burke Quinlan E, Solodkin A, Small SL, Cramer SC. Utility of EEG measures of brain function in patients with acute stroke. J Neurophysiol. 2016;115(5):2399–405. 26936984 10.1152/jn.00978.2015 PMC4922461

[4] Finnigan S, Van Putten MJAM. EEG in ischaemic stroke: quantitative EEG can uniquely inform (sub-)acute prognoses and clinical management. Clin Neurophysiol. 2013;124(1):10–9. 22858178 10.1016/j.clinph.2012.07.003

[5] Johnston PR, McIntosh AR, Meltzer JA. Spectral slowing in chronic stroke reflects abnormalities in both periodic and aperiodic neural dynamics. Neuroimage. 2023;37:103277. 36495856 10.1016/j.nicl.2022.103277 PMC9758570

[6] Dubovik S, Pignat J-M, Ptak R, Aboulafia T, Allet L, Gillabert N, et al. The behavioral significance of coherent resting-state oscillations after stroke. Neuroimage. 2012;61(1):249–57. 22440653 10.1016/j.neuroimage.2012.03.024

[7] Grefkes C, Fink GR. Connectivity-based approaches in stroke and recovery of function. Lancet Neurol. 2014;13(2):206–16. 24457190 10.1016/S1474-4422(13)70264-3

[8] Kancheva I, Van Der Salm SMA, Ramsey NF, Vansteensel MJ. Association between lesion location and sensorimotor rhythms in stroke – a systematic review with narrative synthesis. Neurol Sci. 2023;44(12):4263–89. 37606742 10.1007/s10072-023-06982-8 PMC10641054

[9] Vatinno AA, Simpson A, Ramakrishnan V, Bonilha HS, Bonilha L, Seo NJ. The prognostic utility of electroencephalography in stroke recovery: a systematic review and meta-analysis. Neurorehabil Neural Repair. 2022;36(4–5):255–68. 35311412 10.1177/15459683221078294 PMC9007868

[10] Mainali S, Darsie ME, Smetana KS. Machine learning in action: stroke diagnosis and outcome prediction. Front Neurol. 2021;12:734345. 34938254 10.3389/fneur.2021.734345 PMC8685212

[11] Daidone M, Ferrantelli S, Tuttolomondo A. Machine learning applications in stroke medicine: advancements, challenges, and future prospectives. Neural Regen Res. 2023;19(4):769–73. 10.4103/1673-5374.382228 PMC10664112 37843210

[12] Kayser J, Tenke CE. On the benefits of using surface Laplacian (current source density) methodology in electrophysiology. Int J Psychophysiol. 2015;97(3):171–3. 26071227 10.1016/j.ijpsycho.2015.06.001 PMC4610715

[13] Saes M, Meskers CGM, Daffertshofer A, Munck JC, Kwakkel G, Wegen EEH. How does upper extremity Fugl-Meyer motor score relate to resting-state EEG in chronic stroke? A power spectral density analysis. Clin Neurophysiol. 2019;130(5):856–62. 30902439 10.1016/j.clinph.2019.01.007

[14] Carrera E, Tononi G. Diaschisis: past, present, future. Brain. 2014;137(9):2408–22. 24871646 10.1093/brain/awu101

[15] Ginsberg MD, Bogousslavsky J. Ischemic stroke topographic subtypes. In: Bogousslavsky J, editor. Cerebrovascular disease: pathophysiology, diagnosis, and management. Malden, MA: Blackwell Science; 1998. p. 921–30.

[16] Llinás RR, Ribary U, Jeanmonod D, Kronberg E, Mitra PP. Thalamocortical dysrhythmia: a neurological and neuropsychiatric syndrome characterized by magnetoencephalography. Proc Natl Acad Sci. 1999;96(26):15222–7. 10611366 10.1073/pnas.96.26.15222 PMC24801

[17] Edlow BL, Takahashi E, Wu O, Benner T, Dai G, Bu L, et al. Neuroanatomic connectivity of the human ascending arousal system critical to consciousness and its disorders. J Neuropathol Exp Neurol. 2012;71(6):531–46. 22592840 10.1097/NEN.0b013e3182588293 PMC3387430

[18] Kayser J. Current source density (csd) interpolation using spherical splines-csd Toolbox (version 1.1). New York State Psychiatric Institute: Division of Cognitive Neuroscience, 2009.

[19] Kayser J, Tenke CE. Principal components analysis of Laplacian waveforms as a generic method for identifying ERP generator patterns: I. Evaluation with auditory oddball tasks. Clin Neurophysiol. 2006;117(2):348–68. 16356767 10.1016/j.clinph.2005.08.034

[20] Putten MJAM. The revised brain symmetry index. Clin Neurophysiol. 2007;118(11):2362–7. 17888719 10.1016/j.clinph.2007.07.019

[21] Ramoser H, Muller-Gerking J, Pfurtscheller G. Optimal spatial filtering of single trial EEG during imagined hand movement. IEEE Trans Rehabil Eng. 2000;8(4):441–6. 11204034 10.1109/86.895946

[22] Blankertz B, Tomioka R, Lemm S, Kawanabe M, Muller K-R. Optimizing spatial filters for robust EEG single-trial analysis. IEEE Signal Process Mag. 2008;25(1):41–56.

[23] Lawhern VJ, Solon AJ, Waytowich NR, Gordon SM, Hung CP, Lance BJ. EEGNet: a compact convolutional neural network for EEG-based brain-computer interfaces. J Neural Eng. 2018;15(5):056013. 29932424 10.1088/1741-2552/aace8c

[24] Huang G. Statistical analysis. In: EEG signal processing and feature extraction. Singapore: Springer; 2019. p. 335–75.

[25] Hsu C-W, Lin C-J. A comparison of methods for multiclass support vector machines. IEEE Trans Neural Netw. 2002;13(2):415–25. 18244442 10.1109/72.991427

[26] Huang J, Ling CX. Using AUC and accuracy in evaluating learning algorithms. IEEE Trans Knowl Data Eng. 2005;17(3):299–310.

[27] Kendall F, McCreary E, Provance P. Muscles, testing and function. Med Sci Sports Exerc. 1994;26(8):1070.

[28] Fugl-Meyer AR, Jääskö L, Leyman I, Olsson S, Steglind S. A method for evaluation of physical performance. Scand J Rehabil Med. 1975;7(1):13–31. 1135616

[29] Folstein MF, Folstein SE, McHugh PR. Mini-mental state: a practical method for grading the cognitive state of patients for the clinician. J Psychiatr Res. 1975;12(3):189–98. 1202204 10.1016/0022-3956(75)90026-6

[30] Collin C, Wade D, Davies S, Horne V. The Barthel ADL Index: a reliability study. Int Disabil Stud. 1988;10(2):61–3. 3403500 10.3109/09638288809164103

[31] Mahoney FI, Barthel DW. Functional evaluation: the barthel index. Md State Med J. 1965;14:61–5. 14258950

[32] Wang M, Zhao S-W, Wu D, Zhang Y-H, Han Y-K, Zhao K, et al. Transcriptomic and neuroimaging data integration enhances machine learning classification of schizophrenia. Psychoradiology. 2024;4:005. 10.1093/psyrad/kkae005 PMC11061866 38694267

[33] Sokolova M, Lapalme G. A systematic analysis of performance measures for classification tasks. Inf Process Manag. 2009;45(4):427–37.

[34] Ulanov M, Shtyrov Y. Oscillatory beta/alpha band modulations: a potential biomarker of functional language and motor recovery in chronic stroke? Front Hum Neurosci. 2022;16:940845. 36226263 10.3389/fnhum.2022.940845 PMC9549964

[35] Haynes J-D, Rees G. Decoding mental states from brain activity in humans. Nat Rev Neurosci. 2006;7(7):523–34. 16791142 10.1038/nrn1931

[36] Roy Y, Banville H, Albuquerque I, Gramfort A, Falk TH, Faubert J. Deep learning-based electroencephalography analysis: a systematic review. J Neural Eng. 2019;16(5):051001. 31151119 10.1088/1741-2552/ab260c

[37] Schirrmeister RT, Springenberg JT, Fiederer LDJ, Glasstetter M, Eggensperger K, Tangermann M, et al. Deep learning with convolutional neural networks for EEG decoding and visualization. Hum Brain Mapp. 2017;38(11):5391–420. 28782865 10.1002/hbm.23730 PMC5655781

[38] Haufe S, Meinecke F, Görgen K, Dähne S, Haynes J-D, Blankertz B, et al. On the interpretation of weight vectors of linear models in multivariate neuroimaging. Neuroimage. 2014;87:96–110. 24239590 10.1016/j.neuroimage.2013.10.067

[39] Zhang JJ, Bai Z, Fong KNK. Resting-state cortical electroencephalogram rhythms and network in patients after chronic stroke. J Neuroeng Rehabil. 2024;21(1):32. 38424592 10.1186/s12984-024-01328-7 PMC10902978

[40] Vecchio F, Pappalettera C, Miraglia F, Deinite G, Manenti R, Judica E, et al. Prognostic role of hemispherical functional connectivity in stroke: a study via graph theory versus coherence of electroencephalography rhythms. Stroke. 2023;54(2):499–508. 36416129 10.1161/STROKEAHA.122.040747

[41] Cassidy JM, Wodeyar A, Wu J, Kaur K, Masuda AK, Srinivasan R, et al. Low-frequency oscillations are a biomarker of injury and recovery after stroke. Stroke. 2020;51(5):1442–50. 32299324 10.1161/STROKEAHA.120.028932 PMC7188582

[42] Borghini G, Astolfi L, Vecchiato G, Mattia D, Babiloni F. Measuring neurophysiological signals in aircraft pilots and car drivers for the assessment of mental workload, fatigue and drowsiness. Neurosci Biobehav Rev. 2014;44:58–75. 23116991 10.1016/j.neubiorev.2012.10.003

