# Supplementary material for: Lesion-specific EEG signatures in stroke: a multi-scale framework integrating oscillations, connectivity, and asymmetry for machine learning decoding
Source: J Neuroeng Rehabil. 2026 Mar 31;23:157. doi: 10.1186/s12984-026-01947-2 (PMC13170151; doi:10.1186/s12984-026-01947-2)
Supplement: Supplementary file 1 — (pdf 1110 KB) [file 12984_2026_1947_MOESM1_ESM.pdf]

Supplementary Material

1 Supplementary Results on EEG and Behavioral Associations

Behavioral scores were categorized into subgroups based on predefined thresholds. For the Manual Muscle Test (MMT), grades 0–2 were classified as the low-strength group, whereas grades 3–5 were classified as the good-strength group. For the Mini-Mental State Examination (MMSE), scores < 21 were classified as the cognitively impaired group, and scores ≥ 21 as the cognitively good group. For the Upper limb Fugl-Meyer Assessment (FMA), scores ≥ 20 were defined as the better-functioning group, and scores < 20 as the poorer-function group. In addition, FMA scores were further stratified as follows: ≥ 60 indicating good function, 40–59 indicating moderate impairment, and < 40 indicating severe impairment.

The results indicated that, in MMT, θ band connectivity primarily involved networks linking the central (C) region with parietal (P) and occipital (O) areas. In the α band, the pattern extended to connections between the frontal (F) and affected motor (AM) regions, as well as between the unaffected motor (UM) region and P and O regions. In the β band, associations were mainly confined to connectivity between the F and AM region. The MMSE was predominantly related to θ band connectivity within right-hemispheric regions. Motor evoked potential (MEP) were significantly associated with connectivity between the UM region and O/P regions across all three frequency bands. FMA were primarily influenced by θ band connectivity between the C region and P/O regions. In contrast, ADL scores were linked to more complex α band networks, involving connections between the F region and the rest of regions.

	MMT		MMSE		MEP		FMA		ADL	
FC	p-value	correlation (r)	p-value	correlation (r)	p-value	correlation (r)	p-value	correlation (r)	p-value	correlation (r)
F-LM	0.092	0.225	0.329	0.132	0.195	0.174	0.389	0.116	0.268	0.149
F-C	0.385	0.117	0.376	0.119	0.116	0.210	0.469	0.098	0.353	0.125
F-RM	0.545	0.082	0.472	0.097	0.201	0.172	0.376	0.120	0.960	0.007
F-P	0.129	0.203	0.152	0.192	0.253	0.154	0.103	0.218	0.172	0.183
F-O	0.162	0.188	**0.024**	0.298	0.286	0.144	0.213	0.167	0.718	0.049
LM-C	0.123	0.207	0.870	0.022	0.861	0.024	0.579	0.075	0.230	0.162
LM-RM	0.442	0.104	0.321	0.134	0.498	0.092	0.854	0.025	0.555	0.080
LM-P	0.052	0.259	0.215	0.167	0.348	0.127	0.294	0.141	0.276	0.147
LM-O	0.264	0.150	0.659	0.060	0.604	0.070	0.785	0.037	0.969	0.005
C-RM	0.065	0.246	0.102	0.219	0.070	0.242	0.110	0.214	0.174	0.183
C-P	**0.023**	0.301	**0.036**	0.279	0.239	0.158	**0.009**	0.345	0.116	0.211
C-O	**0.046**	0.265	0.404	0.113	0.322	0.133	**0.028**	0.291	0.052	0.259
RM-P	0.092	0.225	**0.021**	0.305	**0.032**	0.285	0.430	0.107	0.268	0.149
RM-O	0.159	0.189	**0.010**	0.339	0.052	0.259	0.174	0.183	0.660	-0.059
P-O	**0.025**	0.296	0.562	0.078	0.628	0.066	0.145	0.196	0.220	0.165

Table S1 Correlation between Θ band EEG features and behavioral measures; Bold indicates significant associations before correction; none remained significant after correction; FC, fuctional connectivity

	MMT		MMSE		MEP		FMA		ADL	
FC	p-value	correlation (r)	p-value	correlation (r)	p-value	correlation (r)	p-value	correlation (r)	p-value	correlation (r)
F-LM	**0.006**	0.363	0.695	-0.053	0.616	0.068	0.204	0.171	0.016	0.317
F-C	0.068	0.243	0.636	-0.064	0.688	0.054	0.287	0.143	**0.032**	0.284
F-RM	0.129	0.203	0.744	0.044	0.472	0.097	0.071	0.241	**0.023**	0.301
F-P	0.059	0.252	0.590	0.073	0.558	0.079	0.280	0.146	**0.027**	0.293
F-O	0.188	0.177	0.275	0.147	0.547	0.081	0.136	0.200	0.100	0.220
LM-C	0.111	0.213	0.756	-0.042	0.682	-0.055	0.748	0.043	0.160	0.189
LM-RM	0.191	0.176	0.864	-0.023	0.887	0.019	0.936	0.011	0.210	0.169
LM-P	0.117	0.210	0.883	0.020	0.246	0.156	0.712	0.050	0.135	0.200
LM-O	0.206	0.170	0.935	0.011	0.314	0.136	0.923	0.013	0.236	0.160
C-RM	0.177	0.181	0.794	0.035	0.514	0.088	0.403	0.113	0.068	0.243
C-P	0.106	0.217	0.121	0.208	0.789	-0.036	0.140	0.198	0.314	0.136
C-O	0.294	0.141	0.832	0.029	0.306	0.138	0.119	0.209	**0.025**	0.296
RM-P	**0.029**	0.290	0.334	0.130	0.078	0.235	0.346	0.127	0.316	0.135
RM-O	0.079	0.234	0.794	-0.035	**0.021**	0.305	0.101	0.220	0.455	0.101
P-O	**0.042**	0.270	0.325	-0.133	0.201	0.172	0.091	0.226	**0.049**	0.262

Table S2 Correlation between α band EEG features and behavioral measures; Bold indicates significant associations before correction; none remained significant after correction; FC, fuctional connectivity

	MMT		MMSE		MEP		FMA		ADL	
FC	p-value	correlation (r)	p-value	correlation (r)	p-value	correlation (r)	p-value	correlation (r)	p-value	correlation (r)
F-LM	**0.010**	0.338	0.677	-0.056	0.493	0.093	0.104	0.217	0.264	0.150
F-C	0.188	0.177	0.376	-0.119	0.393	0.115	0.273	0.148	0.555	0.080
F-RM	0.482	0.095	0.534	0.084	0.306	0.138	0.226	0.163	0.216	0.166
F-P	0.275	0.147	0.909	0.015	0.530	0.085	0.354	0.125	0.627	0.066
F-O	0.275	0.147	0.928	0.012	0.628	0.066	0.557	0.079	0.542	0.083
LM-C	0.987	0.002	0.624	-0.066	0.053	-0.258	0.499	-0.091	0.805	-0.033
LM-RM	0.066	0.245	0.935	0.011	0.854	0.025	0.835	0.028	0.696	0.053
LM-P	0.257	0.153	0.719	0.049	0.553	0.080	0.718	0.049	0.642	0.063
LM-O	0.257	0.153	0.624	0.066	0.306	0.138	0.936	0.011	0.936	0.011
C-RM	0.701	0.052	1.000	0.000	0.688	0.054	0.841	0.027	0.323	0.133
C-P	0.826	0.030	0.404	0.113	0.887	-0.019	0.866	-0.023	0.636	0.064
C-O	0.404	0.113	0.647	0.062	0.564	0.078	0.354	0.125	0.280	0.146
RM-P	0.167	0.186	0.492	0.093	0.120	0.208	0.748	-0.043	0.800	0.034
RM-O	0.172	0.183	0.394	-0.115	**0.043**	0.269	0.531	0.085	0.736	-0.046
P-O	0.059	0.252	0.261	-0.151	0.456	0.101	0.164	0.187	0.308	0.137

Table S3 Correlation between β band EEG features and behavioral measures; Bold indicates significant associations before correction; none remained significant after correction; FC, fuctional connectivity

2 Supplementary Results: Common Spatial Patterns (CSP) Spatial Patterns

To address the physiological interpretability of the machine-learning results, we visualized the spatial distributions associated with the CSP components for each pairwise classification task and each frequency band (Fig. S1). Here, “CSP spatial patterns” refer to the scalp topographies obtained by transforming the learned CSP spatial filters into forward models (i.e., spatial patterns), which indicate how strongly each EEG channel contributes to the corresponding CSP component in sensor space. Unlike the CSP filters (which are optimized to extract discriminative component time series), the spatial patterns are commonly used for interpretation because they provide a more direct, physiologically meaningful topographical representation of the component [1].

For the comparison between healthy controls and stroke patients (HC vs. P), the CSP topographies are relatively smooth and exhibit a large-scale spatial organization, characterized by a clear anterior–posterior gradient. Specifically, the patient maps show reduced pattern amplitudes over frontal regions and increased amplitudes over posterior (occipito-parietal) regions. This large-scale pattern may reflect stroke-related alterations in global rhythmic state, attention/arousal regulation, and/or cortical excitation–inhibition balance, and is also compatible with broad network reorganization after stroke. Notably, we did not observe a robust, strongly lateralized motor-cortex signature in these CSP patterns.

Comparisons were performed among patient subgroups defined by lesion location: basal ganglia (P1), fronto-temporal/centrum semiovale (P2), and brainstem (P3). Specifically, for the pairwise comparisons (P1 vs. P2, P1 vs. P3, and P2 vs. P3), the CSP topographies appear less stable and more spatially focal, with greater apparent influence from individual electrodes. This observation suggests increased sensitivity to inter-subject variability and potential channel-level noise. We note that CSP-derived components are optimized for discrimination rather than for isolating a single physiological source; consequently, compared with unsupervised decompositions (e.g., independent component analysis), CSP patterns in small and heterogeneous clinical subgroups may be more susceptible to class-specific noise and should be interpreted cautiously. We therefore provide these CSP topographical maps in the Supplementary Material to support transparency and interpretation, while avoiding overinterpretation of fine-grained spatial details in the main text.

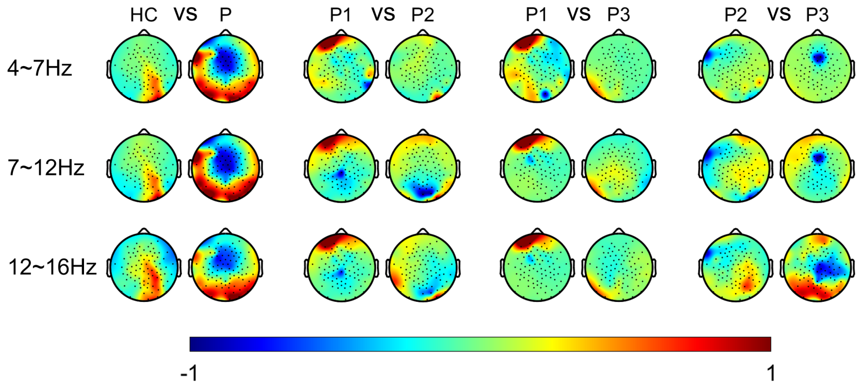

Fig. S1 Topographical representations of CSP spatial patterns for the θ (4–7 Hz), α (7–12 Hz), and β_1 (12–16 Hz) bands. Columns correspond to pairwise classifications between healthy controls (HC) and patients (P), as well as between patient subgroups (P1, P2, and P3). Pattern amplitudes were normalized for visualization only.

References

[1] Haufe, S., Meinecke, F., Görgen, K., Dähne, S., Haynes, J.-D., Blankertz, B., Biessmann, F.: On the interpretation of weight vectors of linear models in multivariate neuroimaging. NeuroImage **87**, 96–110 (2014)
